# *Yersinia pestis* Survival and Replication in Potential Ameba Reservoir 

**DOI:** 10.3201/eid2402.171065

**Published:** 2018-02

**Authors:** David W. Markman, Michael F. Antolin, Richard A. Bowen, William H. Wheat, Michael Woods, Mercedes Gonzalez-Juarrero, Mary Jackson

**Affiliations:** Colorado State University, Fort Collins, Colorado, USA (D.W. Markman, M.F. Antolin, R.A. Bowen, W.H. Wheat, M. Gonzalez-Juarrero, M. Jackson);; Burrell College of Osteopathic Medicine, Las Cruces, New Mexico, USA (M. Woods)

**Keywords:** Plague, *Yersinia* *pestis*, coccobacillus, epizootic, feral macrophage, disease reservoir, ameba, amoeba, amoebae, *Dictyostelium* *discoideum*, slime mold, eukaryote, ecology, biological evolution, endemic, trophozoite, phagocytosis

## Abstract

Plague ecology is characterized by sporadic epizootics, then periods of dormancy. Building evidence suggests environmentally ubiquitous amebae act as feral macrophages and hosts to many intracellular pathogens. We conducted environmental genetic surveys and laboratory co-culture infection experiments to assess whether plague bacteria were resistant to digestion by 5 environmental ameba species. First, we demonstrated that *Yersinia pestis* is resistant or transiently resistant to various ameba species. Second, we showed that *Y. pestis* survives and replicates intracellularly within *Dictyostelium discoideum* amebae for ˃48 hours postinfection, whereas control bacteria were destroyed in <1 hour. Finally, we found that *Y. pestis* resides within ameba structures synonymous with those found in infected human macrophages, for which *Y. pestis* is a competent pathogen. Evidence supporting amebae as potential plague reservoirs stresses the importance of recognizing pathogen-harboring amebae as threats to public health, agriculture, conservation, and biodefense.

The etiologic agent of plague, *Yersinia pestis*, is a gram-negative coccobacillus and a facultative intracellular pathogen. *Y. pestis* exhibited the highest overall mortality rate of any infectious disease from its earliest recorded emergence through 1941 (*1*). During 2010–2015, a mean of 650 cases were reported globally each year, with a case fatality rate of 23%–41% (depending on manifestation as bubonic, pneumonic, or septicemic plague), rising to 66%–100% when adequate medical care was not promptly received (*2*). *Y. pestis* primarily infects small ground-dwelling mammals, specifically of the taxonomic order Rodentia, but maintains high spillover potential to other vertebrates, including humans, caused by its high virulence and fleaborne transmission. Epizootic plague is typically vectored by multiple flea species and is transmitted within and between meta-populations of hosts by flea bites (Figure 1).

**Figure 1:**
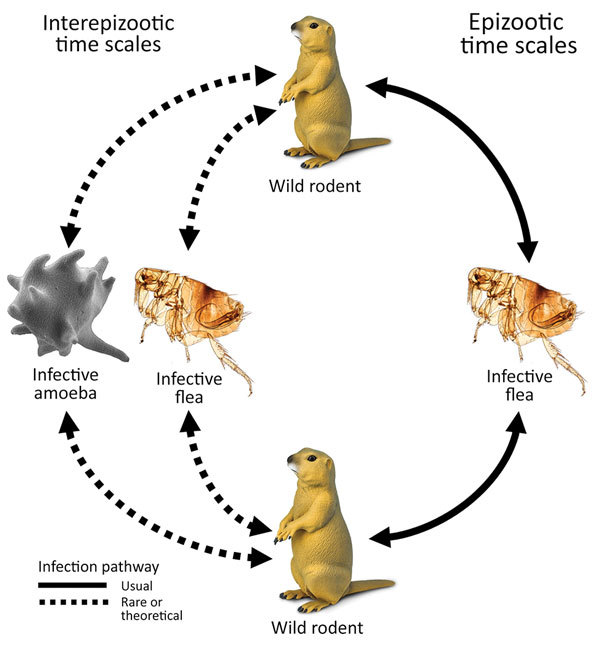
Infection pathways for plague. During plague epizootics, transmission occurs through flea vectors within meta-populations of ground-dwelling rodents. It is unknown by what route or mechanism *Yersinia pestis* is maintained during interepizootic periods of plague quiescence. Previous research on fleas has not strongly supported their reservoir potential across interepizootic periods (*3*). The experiment and analysis of this study test the hypothesis that amoeboid species demonstrate reservoir potential for *Y. pestis*. If *Y. pestis* is maintained within ameba reservoirs, we suspect that epizootic recrudescence may occur when infected soilborne amebae enter the bloodstream of naive rodent hosts (by entering wounds from antagonistic host-to-host interactions or burrowing activities). Amebae typically lyse when incubated at 37°C and simultaneously release their intracellular cargo, potentially initiating an infection.

Plague ecology is characterized by sporadic epizootics, followed by 2–5-year cryptic dormancy periods (*3**–**9*). Despite much information on epizootic transmission mechanisms, little is known about the origin of re-emergent plague cases in wild animal populations (Figure 1). Plague among wild animals commonly re-emerges in plague foci after multiple years of inactivity, despite ongoing biosurveillance and attempts at detection during interepizootic periods. The existence of environmental plague reservoirs has been theorized for >80 years (*3**–**13*). Various avenues of recent research suggest that soil-dwelling amebae may be competent environmental reservoirs of *Y. pestis*. Amebae are a taxonomically diverse group of phagocytic organisms residing in every major lineage of eukaryotes. Amebae are pervasive in soil and water environments and are recognized for their ability to harbor pathogens that drastically affect ecologic communities (*14**–**19*). Free-living amebae cycle between 2 distinct life-states: trophozoites, an active, mobile, feeding state; and cysts or spores, a robust dormant state induced in part by adverse environmental conditions.

Ameba reservoir potential for *Y. pestis* is indicated by 4 major factors: the ability of related *Y. enterocolitica* and *Y. pseudotuberculosis* bacteria to persist in protozoan amebae (*20**–**22*); correlative data indicating plague epizootics temporally follow periods of increased precipitation known to reanimate ameba cysts (*5**,**23**,**24*); the demonstrated ability of *Y. pestis* to express various proteins enabling escape of the phagolysosome in a diverse array of phagocytic cells including human macrophages (*25**–**27*); and prior associations between *Y. pestis* and the soil amebae, *Vermamoeba* (formerly *Hartmanella*) *rhysodes* and *Acanthamoeba castellanii,* that demonstrate intracellular persistence up to 5 days (*13**,**28**,**29*). Amebae display a high degree of functional homology with mammalian macrophages, leading to the description of amebae as feral macrophages. The ameba reservoir hypothesis is compelling for many pathogens with unexplained sporadic occurrence and cryptic dormancy periods as supported by a growing catalog (>225) of intracellular pathogens capable of surviving and/or replicating within amebae under diverse conditions (*14**,**17**,**18**,**30*).

We tested the hypothesis that 5 species of environmentally ubiquitous amebae demonstrate reservoir potential for the maintenance of *Y. pestis*. We implemented field and laboratory investigations to assess environmental co-occurrence of study ameba species with plague epizootics; experimental infection prevalence in amebae; experimental infection intensity; intraameba bacterial location; bacterial viability postphagocytosis; and bacterial replication inside trophozoite amebae. We discuss the potential for *D. discoideum* ameba to act as interepizootic reservoirs, the functional homology between phagocytic amebae and mammalian macrophages, and the ability of ameba to exert selective pressure on the evolutionary trajectory of pathogen virulence and transmission mode. Further, we stress the importance of recognizing pathogen-harboring amebae as potential threats to global health, agriculture, conservation, and biodefense.

## Materials and Methods

By using field experiments, we molecularly assessed the co-occurrence of amebae and *Y. pestis* in prairie dog burrows in the Pawnee National Grassland of northeastern Colorado, USA. This grassland is an established plague foci that has exhibited recurrent plague epizootics since ≈1940 (*31*). We used molecular analyses of soil and amebae cultured from the soil to identify candidate ameba species that may act as reservoirs for plague persistence.

### Plague-Endemic Soil Isolates

We selected 24 prairie dog burrows from 8 prairie dog colonies, which can contain hundreds of animals, on the basis of suspected plague presence indicated by sustained decreases in population size during a 3-week observation period in August 2016 (Technical Appendix Figure 1). We selected individual burrows within the colony boundaries on the basis of apparent prairie dog activity (feces, freshly excavated soil, and noncollapsed burrow structure) and along a gradient from the center of the colony to the periphery. We collected soil by attaching 50-mL conical tubes to a 6-m flexible metal probe, maneuvering the probe into the prairie dog burrow to maximum achievable depth, and using the probe to scrape soil into the tubes. We sealed viable soil samples (>20 mL from >3 m deep) and stored them at 22°C until processing within 12 hours.

### Cultivation of Amebae from Soil

 We isolated amebae from soil in plague-affected prairie dog burrows by using modified culture methods (*32*) (Technical Appendix Figure 2), incubated culture plates at 28°C, and observed for changes daily. We supplemented liquid medium with gentamicin (200 μg/mL) after 72 hours or at earliest detection of any bacterial growth. We aseptically transferred ameba cultures without bacterial contamination to 25-cm^2^ tissue culture flasks in ameba-specific media containing penicillin/streptomycin. We identified ameba by using multiplex and endpoint PCR after extracting DNA by using a QIAGEN DNeasy Blood & Tissue Kit ( QIAGEN, Hilden, Germany) (*33**,**34*) (Technical Appendix Figure 3).

### Bacterial Strains and Culture Conditions

We cultured *Y. pestis* strains from frozen stocks in lysogeny broth (LB) medium. We used 2 strains of *Y. pestis* throughout the study: a nontransformed prototypical strain of *Y. pestis* CO92 and a recombinant *gfp*-expressing strain, *Y. pestis* CO92 *pgm*+, pCD1, pGFPuv, amp+, from the Centers for Disease Control and Prevention (Fort Collins, CO, USA). We cultured the transformed strain by using 100 μg/mL carbenicillin to maintain selective pressure for retention of *gfp* plasmids. Culture conditions simulated a mammalian host environment (37°C for 24 h to stationary phase) and then an extra-host environment (28°C for 24 h) to activate phenotypically plastic expression profiles. We monitored bacterial growth spectrophotometrically at OD600.

### Ameba Strains and Culture Conditions

We obtained stocks of *A. lenticulata* (ATCC 30841), *A. castellanii* (ATCC 30234), *A. polyphaga* Linc-Ap1 (CCAP 1501/18), and *V. vermiformis* (ATCC 50237) from the American Type Culture Collection (Manassas, VA, USA) and the Culture Collection of Algae and Protozoa (https://www.ccap.ac.uk/) and *Dictyostelium discoideum* (NC4A2) from DictyBase (http://dictybase.org/). We axenically cultivated ameba stocks with genera-specific media in T25 tissue culture flasks at 28°C and verified them to be axenic by using standardized methods (*19**,**35**–**37*).

### Co-culture Experiments

#### Intraameba Infection Prevalence and Intensity Assays

We individually co-cultured laboratory ameba species with *Y. pestis* by using established methods (*21*). We adjusted viable ameba trophozoite densities to 5 × 10^5^ trophozoites/mL in triplicate 25-cm^2^ tissue culture flasks and combined *Y. pestis* (CO92 *pgm*+, pCD1, pGFPuv, amp+) cultures with ameba flasks (excluding ameba controls), resulting in 5 × 10^7^ viable *Y. pestis* cells/mL and a multiplicity of infection (MOI) of 100 on the basis of OD 600 calculations. We incubated co-cultures at 28°C for 4 h before removing infected amebae, ameba controls, and bacteria controls from the surface of the flasks and washing them 3 times with Page amoeba saline (PAS) at 100 × *g* for 5 min (*36*). We then exposed amebae to gentamicin (100 μg/mL) for either 1 or 4 h to eliminate residual extracellular bacteria, then washed them 3 more times to remove antibiotic drug residue. Finally, we removed the supernatant from the last wash, concentrated it via centrifugation (4,400 × *g* for 10 min), then plated it on LB agar to ensure no extracellular bacteria persisted.

We fixed aliquots of each infected ameba treatment in 4% paraformaldehyde for 15 min before washing (4,400 × *g*, 5 min) and resuspending in 1× PAS for microscopic analysis. We used a fluorescent confocal microscope (Zeiss LSM 510 with ZEN 2009 SP2 software [Carl **Zeiss** GmbH, Thornwood, NY, USA]) to determine mean infection prevalence (the percentage of amebae containing >1 intracellular *Y. pestis* bacterium across 16 fields of view per treatment replicate). We determined mean infection intensity and its distribution by quantifying the number of intracellular bacteria per individual infected ameba, verified by z-stack confocal microscopy across 16 fields of view per treatment replicate. We used 1-way measured analysis of variance (ANOVA) on prevalence and intensity means across all 5 amebae species. We log-transformed data as necessary to meet model assumption and used least-squared mean analyses with Tukey’s adjustments for pairwise comparisons.

#### Ultrastructural Description of Intraameba Bacterial Location

We used *Y. pestis* (CO92 *pgm*+, pCD1, pGFPuv, amp+) in similar co-culture infection assays with *A. castellanii* (MOI 100 in PAS at 28°C). We co-cultured bacteria for durations of 10 min, 30 min, and 24 h to assess proximal and delayed effects of phagocytosis on bacterial cell viability and intraameba bacterial location. After co-culture, mixtures were fixed in standard electron microscopy fixative for 2 h, then washed 2 times in 0.1 M cacodylate buffer. We then shipped fixed samples in 0.1 M cacodylate buffer to the Cryo-electron Microscopy Laboratory at the University of Texas Medical Branch (Galveston, TX, USA) for transmission electron microscopy (TEM) according to standardized procedures. We determined bacterial location within amebae by ultrastructural analysis of transmission electron micrographs and studied intracellular bacterial morphology to assess ameba-mediated bacterial lysis as measured by cell size, shape, and apparent membrane integrity.

#### Intra-meba Bacterial Survival and Quantification of Intraameba Bacterial Replication

We performed intraameba bacterial survival and replication assays in triplicate across 5 ameba species (*A*. *castellanii, A.* *lenticulata, A.* *polyphaga, D.* *discoideum*, and *V.* *vermiformis*); 2 bacteria species (*Y*. *pestis* CO92 and *Escherichia coli*); 5 postinfection time points (0, 1, 4, 24, and 48 h); and 3 antibiotic drug exposure periods (0, 1, and 4 h) for removing extracellular bacteria postinfection. We used *E. coli* as an ameba-susceptible control bacterium. We performed co-cultures in 200-μL volumes within 96-well plates at a MOI of 100 in 1/2× dilute ameba growth medium at 28°C for 1 h and used control ameba and bacteria as monocultures. After initial infection, we removed the supernatant of each well, washed wells 3 times with PAS, exposed them to gentamicin (100 μg/mL), washed 3 times more, and incubated them in PAS. PAS was verified to be bacteriostatic to *Y. pestis*, thereby precluding extracellular bacterial replication and continuous ingestion by amebae. We lysed infected ameba trophozoites selectively with 100 μL 0.5% sodium deoxycholate for 5 min before serially diluting and plating on LB agar. We incubated plates at 28°C for 48 h before counting CFUs to determine bacterial survival and replication after phagocytosis by amebae. The 0.5% sodium deoxycholate lysing detergent had no effect on CFU counts in bacterial monoculture controls (data not shown).

## Results

*Y*. *pestis* and 5 species of amebae co-occur in soils of prairie dog burrows undergoing plague epizootics. We cultured a wide diversity of amebae from soil within plague-positive prairie dog burrows in northeastern Colorado and identified live amebae of each study species (Technical Appendix Figure 3). Among 8 prairie dog colonies, 24 burrows sampled yielded 15 heterogeneous ameba cultures free of bacteria or fungi. We identified >1 *Acanthamoeba* spp. From 86.6% of cultures (n = 13), *D. discoideum* from 53.3% of cultures (n = 8), and *V. vermiformis* from 6.6% of cultures (n = 1).

**Figure 2:**
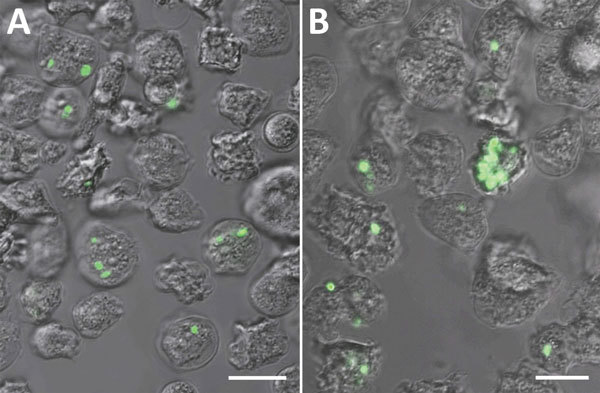
Representative fluorescent confocal images of (A) *Acanthamoeba castellanii* (B) and *Dictyostelium discoideum* after experimental co-culture with *Yersinia pestis* (CO92 *pgm*+, pCD1, pGFPuv, amp+) and removal of extracellular bacteria. After co-culture of ameba trophozoites and *Y. pestis*, we determined the prevalence and intensity of bacterial uptake by manual counting of amebae by using z-stack fluorescent confocal microscopy and averaging across 15 fields per replicate of each ameba species. Confocal count data represent the minimum prevalence/intensity values. Bacteria adherent to the outside of ameba or those with uncertain intracellular status were discarded. The minimum count threshold to reduce random count bias to accepted levels was determined to be 500 per ameba species. Scale bars indicate 30 μm.

*Y. pestis* is phagocytosed by amebae with heterogeneous prevalence and intensity. Representative fluorescent confocal micrographs of *A. castellanii* and *D. discoideum* illustrate differences in infection intensity and prevalence (Figure 2). ANOVA F-test results indicate significant differences in infection prevalence (or phagocytic efficiency) among ameba species (p = 0.0231) (Table). Repeat experiments maintained relative ranking of mean infection intensity and infection prevalence across ameba species (*A. castellanii*, n = 1,441; *A. lenticulata*, n = 1,156; *A. polyphaga*, n = 737; *D. discoideum*, n = 624; and *V. vermiformis*, n = 528). Pairwise comparisons indicate *V. vermiformis* has significantly lower infection prevalence than *A. lenticulata* (p = 0.0344). Infection prevalence ranged from 24.07% in 1 replicate of *V. vermiformis* to 54.83% in 1 replicate of *A. lenticulata.*

**Table:** Properties and kinetics of 5 amebae species after experimental *Yersinia pestis* infection

Species	Dormant state	Infection prevalence*			Infection intensity†			Intracellular survival‡					Intracellular replication
								24 h			48 h		
		Mean, %	SEM		Mean, %	SEM		Mean, %	SEM		Mean, %	SEM	
*Acanthamoeba castellanii*	Cyst	33.63	5.21		4.22	0.61		0	0		0	0	Inconclusive§
*A. lenticulata*	Cyst	51.66	3.17		6.41	0.43		10	11.55		0	0	No
*A. polyphaga*	Cyst	49.08	5.41		5.36	0.37		31.66	22.04		0	0	No
*Dictyostelium discoideum*	Spore	39.24	3.13		3.57	0.97		270	19.92		226.67	22.71	Yes
*Vermamoeba vermiformis*	Cyst	29.61	3.4		1.84	0.13		10	9.66		0	0	No

*Mean percentage of amebae containing >1 intracellular bacterium. †Mean no. of intracellular bacteria per individual infected ameba. ‡Mean no. of surviving intracellular bacteria (relative to control) in experiments with 1 and 4 h of antibiotic drug exposure. §We observed no replication in the intraameba replication assay, which we used to count intra-ameba bacterial colony-forming units before and after co-culture. However, we observed probable but nondefinitive mitotic bacterial replication in the TEM micrographs (Figure 4, panel A).

**Figure 3:**
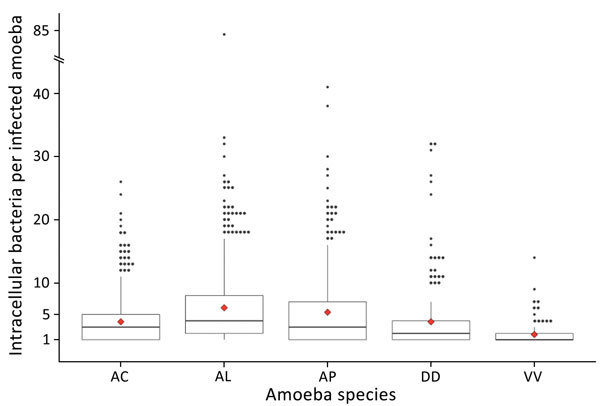
Boxplots of infection intensity across ameba species after experimental infection with *Yersinia pestis*. Infection intensity frequencies followed a strong negative binomial distribution. Median infection intensities (horizontal lines inside boxes): AC = 3, AL = 4, AP = 3, DD = 2, VV = 1. Red diamonds denote mean infection intensity (Table). Each ameba species had several high-intensity outliers ranging up to a maximum of 84 intracellular bacteria observed in 1 *A. lenticulata* ameba (note broken y-axis). AC, *Acanthamoeba castellanii* (n = 1,441); AL, *A. lenticulata* (n = 1,156); AP, *A. polyphaga* (n = 737); DD, *Dictyostelium discoideum* (n = 624); VV, *Vermamoeba vermiformis* (n = 528).

Infection intensity was also significantly different among ameba species (p = 0.0014) (Table). Pairwise comparisons showed *V. vermiformis* has a significantly lower infection intensity than both *A*. *lenticulata* (p = 0.0014) and *A*. *polyphaga* (p = 0.0082) and that *D*. *discoideum* has a significantly lower infection intensity than *A*. *lenticulata* (p = 0.0455). These findings demonstrate genus-level differences in infection intensity. Infection intensity frequencies followed a strong negative binomial distribution (Figure 3). Each ameba species had several high-intensity outliers ranging up to a maximum of 84 intracellular bacteria observed in 1 *A*. *lenticulata* ameba (Figure 3).

**Figure 4:**
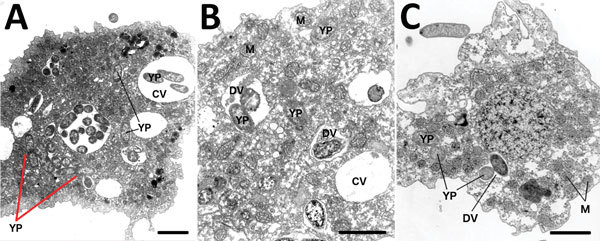
Representative transmission electron micrographs (TEM) depict *Acanthamoeba castellanii* amebae during A) 10-minute, B) 30-minute, and C) 24-hour co-cultures (multiplicity of infection 100) with *Yersinia pestis* (CO92 *pgm*+, pCD1, pGFPuv, amp+). Red arrows in panel A indicate potential intraameba mitotic division of *Y. pestis* bacterium. Visual analysis of TEM micrographs proved inconclusive for identifying the bacterial division septum. *Y. pestis* resides within the potential replicative niche of a tight-fitting vacuolar membrane, similar to *Yersinia*-containing vacuoles observed in macrophages. YP, *Y. pestis*; CV, central vacuole; DV, digestive vacuole; M, mitochondria. Scale bars indicate 3 μm.

*Y*. *pestis* resides in digestive and central vacuoles of both *D*. *discoideum* and *A*. *castellanii* amebae. Green fluorescent protein expressed by intracellular *Y*. *pestis* co-localizes with ameba vacuoles (Figure 2). TEM micrographs depict intracellular *Y. pestis* maintaining cellular shape and apparent membrane integrity inside *A. castellanii* ameba for <24 h postinfection (Figure 4). Ultrastructural analysis of TEM images reveals *Y. pestis* persistence within the niche of a tight-fitting vacuolar membrane visually similar to *Yersinia*-containing vacuoles (YCVs) observed in infected macrophages (*27*).

**Figure 5:**
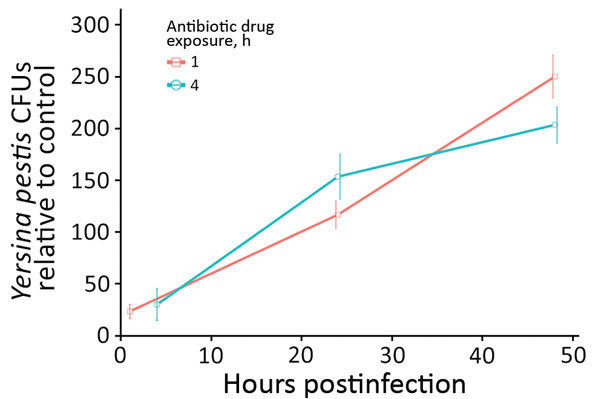
Intraameba *Yersinia pestis* abundance in *Dictyostelium discoideum* across 2 postinfection antibiotic drug exposure periods, 1 hour and 4 hour. In *D. discoideum*, the abundance of viable intracellular *Y. pestis* was significantly greater at each successive time point (24 and 48 hours) after the 1-hour antibiotic drug treatment (p = 0.01 and p = 0.002, respectively). After the 4-hour antibiotic drug treatment in *D. discoideum*, the abundance of viable intracellular *Y. pestis* at 24 and 48 hours was significantly greater than at 4 hours (p = 0.008 and p = 0.001, respectively). The abundance of viable *Y. pestis* within *D. discoideum* at 48 hours postinfection was not significantly different between the 1-hour and 4-hour antibiotic drug treatments (p = 0.1624). Viable intracellular *Y. pestis* abundance was significantly greater in *D. discoideum* compared with all other species at 48 hours postinfection (p<0.001).

*Y. pestis* can survive inside *D. discoideum* amebae for >48 hours, but we found genus-level differences in intraameba survival of *Y*. *pestis* (Table). The bacterium did not survive beyond 24 h postinfection in *A*. *castellanii*, *A*. *lenticulata, A*. *polyphaga*, or *V*. *vermiformis*. However, *Y*. *pestis* co-cultured with *D*. *discoideum* exhibited consistent intracellular survival for >48 h postinfection under variable treatment conditions (Table; Figure 5). *Y*. *pestis* exhibited significantly higher survival/recoverability when co-cultured with amebae as compared to *Y. pestis* monoculture controls (p<0.001). *Y*. *pestis* monoculture controls yielded a mean of 17 CFUs after 1 h of gentamicin exposure with no recoverable control bacteria across all other treatments. Conversely, *E*. *coli* bacteria did not significantly persist under any treatment conditions when co-cultured with ameba (p<0.001). Uninfected ameba control lysates consistently yielded zero bacteria across all ameba species and treatments (data not shown). All *Y. pestis* co-cultures exposed to antibiotics had supernatants free of extracellular bacteria. *Y. pestis* had no cytopathic effect on any of the tested ameba species as verified by comparing ameba abundance between co-culture treatments and ameba monoculture controls (data not shown).

*Y*. *pestis* replicates intracellularly in *D. discoideum* amebae for >48 hours postinfection (Table; Figure 5). In *D. discoideum*, the abundance of viable intracellular *Y. pestis* was significantly greater at each successive time point (24 and 48 h postinfection) after 1 h of antibiotic drug exposure (p = 0.01 and p = 0.002, respectively). Additionally, the abundances of viable *Y. pestis* in *D. discoideum* at 24 and 48 h postinfection were significantly greater than immediately after the 4-h antibiotic treatment (p = 0.008 and p = 0.001, respectively). After 48 h postinfection, viable intracellular *Y. pestis* was only recovered from *D. discoideum* treatments. Because the data did not meet standard ANOVA assumptions of normality despite transformation attempts, we used a nonparametric Kruskal-Wallis rank-sum test to compare treatment means by species. Results indicated that the increased abundance of *Y. pestis* in *D. discoideum* was significant compared with all other species at 48 h postinfection (p<0.001).

## Discussion

We demonstrate that *Y. pestis* (CO-92) can survive and replicate intracellularly within the social, heterogamous ameba *D. discoideum*, whereas *Y. pestis* is only transiently resistant to 4 species of free-living and cyst-forming amebae (*A. castellanii, A. lenticulata, A. polyphaga, and V. vermiformis*). Relative to *E. coli* controls, *Y. pestis* demonstrated significantly increased survival and replication within amebae despite the 4 cyst-forming amebae successfully killing the bacteria by using unidentified mechanisms.

Amebae cultured from soil in prairie dog colonies with active plague epizootics confirm that ameba species used in our experiments co-occur spatially and temporally with *Y. pestis* under natural conditions. Interactions between amebae and *Y. pestis* could select for increasingly ameba-resistant phenotypes, considering the transient resistance already observed in 4 cyst-forming ameba species. Other research has demonstrated the potential for amebae to affect pathogen transmission mode, alter virulence, and act as training grounds for intracellular pathogens by selecting for traits enabling macrophage invasion or avoidance (*17**,**38*).

Genus-level differences in ameba infection intensity and infection prevalence confirm that various ameba species have greater reservoir potential than others. In accordance with super-spreader theories, a minority of individual ameba harboring atypically high numbers of intracellular bacteria may be disproportionately causative for pathogen maintenance and re-emergence.

We observed a shorter duration of survival for *Y. pestis* in *A. castellanii* compared with prior experiments (24 hs vs. 5 d in *13*), likely from differing co-culture conditions and ameba strains. Incubation temperatures differed between this and prior experiments (28°C vs. 4°C and 25°C in *13*). Many *Y. pestis* virulence factors are temperature regulated and may differentially facilitate cellular invasion, inhibition of the phagolysosomal pathway, and intracellular persistence (*1**,**17**,**38*). Additionally, *A. castellanii* (ATCC 30234) used in this study was originally derived from yeast cultures in London in 1930, whereas *A. castellanii* (ATCC 30010), used by Benavides-Montaño et al. (*13*), was originally isolated from California soil in 1957 and enabled longer intracellular survival of *Y. pestis*. Intracellular survival may be affected by traits acquired by co-evolution between amebae and resistant bacteria in soil environments (*17*).

In macrophages, *Y. pestis* recruits host Rab1b protein to the phagosome, resulting in inhibition of phagosome acidification and disruption of the remaining phagolysosomal metabolic pathway (*26**,**27**,**39**–**41*). *Y*. *pestis* then establishes a replicative niche within the YCV, characterized by a tight-fitting vacuole that expands commensurately with bacterial replication (*27*). Examination of TEM micrographs shows that intracellular bacteria are localized within form-fitting vacuolar membranes, similar to the YCVs found in macrophages (Figure 4).

The successful intracellular survival of *Y. pestis* in *D. discoideum* for >48 h demonstrates that *Y. pestis* is an ameba-resistant bacterium. This classification supports the potential for *D. discoideum* or related ameba species to be environmental reservoirs of plague. Intracellular survival of the observed duration is consequential given that typical interactions between bacteria and phagocytic cells result in bacterial death in <40 min (*27*). Most phagocytosed bacteria cannot survive digestive processes characteristic of phagocytic cells including phagolysosome fusion and acidification, or the subsequent recruitment of endosomal lytic factors (*26**,**27**,**41*). Ongoing research assesses the maintenance of viable *Y. pestis* through the entire *D. discoideum* life cycle, including transmissible dormant spores.

Ameba-resistant pathogens often replicate in vacuoles before escaping into the cytosol or outside of the phagocytic cell entirely. In addition to viable intracellular persistence, we observed active intracellular replication of *Y. pestis* (CO-92) in *D. discoideum* (Figure 5) and possible, but unconfirmed, replication of *Y. pestis* (CO92 *pgm*+, pCD1, pGFPuv, amp+) in *A. castellanii* (Figure 4, panel A). Analysis of TEM micrographs proved inconclusive for identifying the bacterial division septum; thus, only *D. discoideum* has conclusively demonstrated intracellular replication of *Y. pestis*. *Y. pestis* CFUs recovered from within *D. discoideum* increased significantly (p = 0.001–0.01; Figure 5) in successive postinfection time points across both antibiotic treatment conditions (except in 1 instance where *Y. pestis* increased nonsignificantly [p = 0.1624; Figure 5]). The consistent absence of extracellular bacteria in all *D. discoideum* co-cultures indicates resistance to digestion and the exploitation of an intraameba replicative niche.

Intracellular replication of *Y. pestis* in macrophages occurs within YCVs, and the formation of YCVs requires metabolic pathway inhibition by recruitment of Rab1b GTPases. Orthologous mechanisms are likely the cause for observed *Y. pestis* replication and survival within amebae. We searched for macrophage Rab1b by using BLAST (http://blast.ncbi.nlm.nih.gov/Blast.cgi) against full genome sequences of each study ameba species and located homologous genetic sequences (99.8% similarity) within *A. castellanii* and *D. discoideum* (GenBank accession nos. XM_004347056.1 and XM_637217.1, respectively [*42**,**43*]). Future research should attempt to establish whether these ameba sequences are functionally orthologous to those identified in macrophages and whether the presence of particular host GTPases is diagnostic of ameba permissiveness to intracellular bacteria.

Results of this study support the reservoir potential of environmental ameba but do not definitively prove that this mechanism occurs in situ. Further research is necessary to determine if the maximum duration of intraameba *Y. pestis* survival corresponds with the durations of cryptic interepizootic persistence that are characteristic of plague dynamics. Increasing evidence for dormant or viable but nonculturable forms of *Y. pestis* may provide explanations underlying hypothesized multiyear survival in ameba spores or cysts (*12**,**25**,**44**–**46*). Outcomes of this research prompt questions regarding evolutionary selection imposed by amebae on environmental pathogens and applications of the ameba reservoir model for other cryptic environmental pathogens. Further research into ameba-mediated pathogenesis and persistence will offer practical insights for public health, conservation, agricultural management, and biodefense.

In conclusion, the mechanisms underlying plague re-emergence following dormancy have eluded researchers for centuries (*1**,**11*). Plague persistence within soilborne microorganisms has been hypothesized as an elusive maintenance mechanism (*6**,**11**,**12**,**25*). We demonstrated spatiotemporal co-occurrence of plague bacterium and various ameba species during an active plague epizootic. Further, we observed the persistence of viable and replicative *Y. pestis* in *D. discoideum* amebae for ˃48 hours postinfection and persistence of *Y. pestis* in 4 cyst-forming ameba species for <24 hours postinfection, whereas ameba-susceptible control bacteria were eliminated by amebae in <1 hour. Thus, *Y. pestis* are respectively ameba-resistant and transiently ameba-resistant under the tested infection conditions. *Y. pestis* bacteria resided within ameba structures that were visually analogous to YCVs observed in infected macrophages. These results encourage research into the eco-evolutionary interactions between pathogenic bacteria, amebae, and host immune factors. The reservoir potential of amebae and their shared infection-permissiveness with phagocytic macrophages show promise in explaining the cryptic properties underlying interepizootic plague transmission and persistence.

Technical Appendix Additional details on study of *Yersinia pestis* infectivity in 5 amebae species.
