# Supplementary material for: Yersinia pestis Survival and Replication in Potential Ameba Reservoir
Source: Emerg Infect Dis. 2018 Feb;24(2):294–302. doi: 10.3201/eid2402.171065 (PMC5782900; doi:10.3201/eid2402.171065)
Supplement: Technical Appendix — Additional details on study of Yersinia pestis infectivity in 5 amebae species. [file 17-1065-Techapp-s1.pdf]

Article DOI: https://doi.org/10.3201/eid2402.171065

Yersinia pestis Survival and Replication in Potential Ameba Reservoir

Technical Appendix

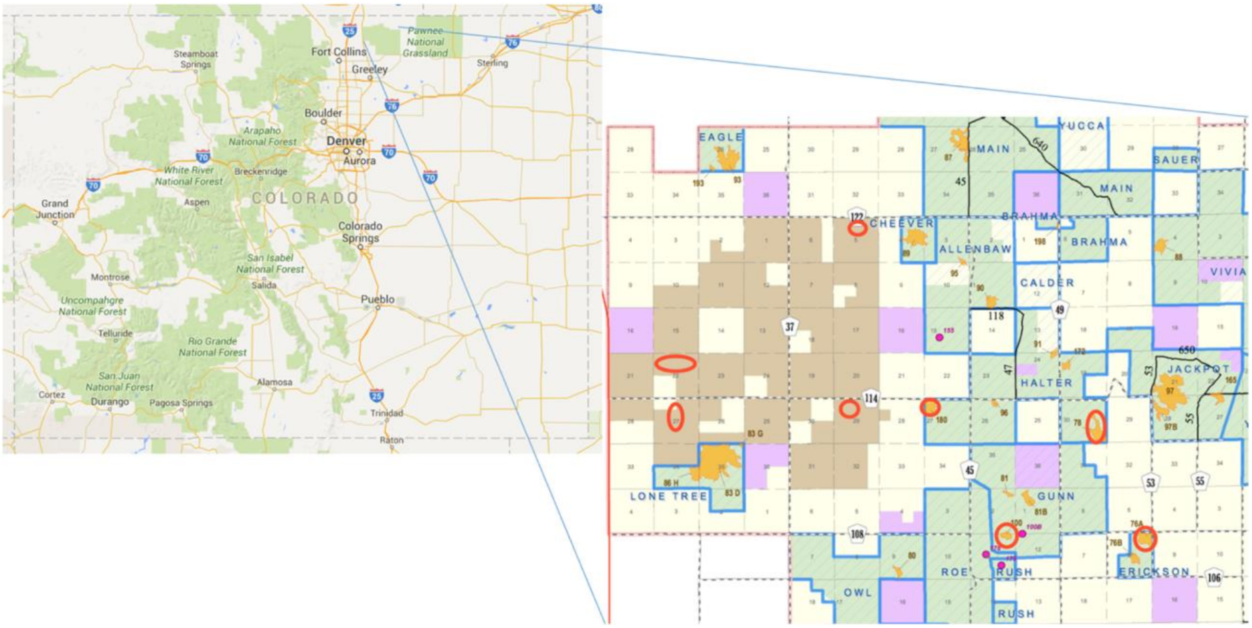

Technical Appendix Figure 1. Pawnee National Grassland, Weld County, Colorado, US. Red circles indicate the burrows of 8 prairie dog colonies where plague epizootics were identified during 2015 and 2016 (*1*). Amebae were cultured from soil samples and identified to species by multiplex and endpoint PCR assays.

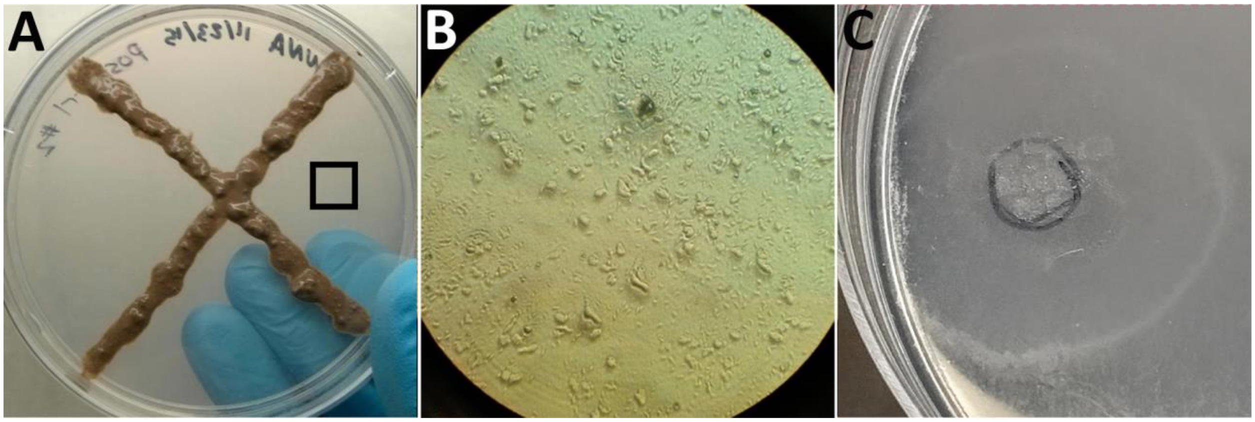

Technical Appendix Figure 2. Amebae isolated from soil samples by using modified methods (*2*). A) Soil obtained from within a prairie dog burrow with an ongoing plague outbreak plated on ameba isolation agar that was pre-coated with heat-killed *Escherichia coli*. Black square indicates region of plate depicted at higher magnification in 2B. B) Trophozoite amebae demonstrate faster motility than most soil microorganisms with the exception of fungal hyphae proliferation. Amebae migrate away from the soil and associated contaminants while digesting the *E*. *coli* spread across the agar surface. Amebae are characterized by their irregular shape with a large internal vacuole. Other diverse soil microorganisms are present on initial isolation plates. C) Ameba isolation agar depicting the migration of amebae and clearance of *E. coli* lawn. Black circle indicates where amebae excised from 2B were re-plated to support further purification by migration. Not pictured are the transfer and acclimation of amebae to liquid cultures in genera specific media.

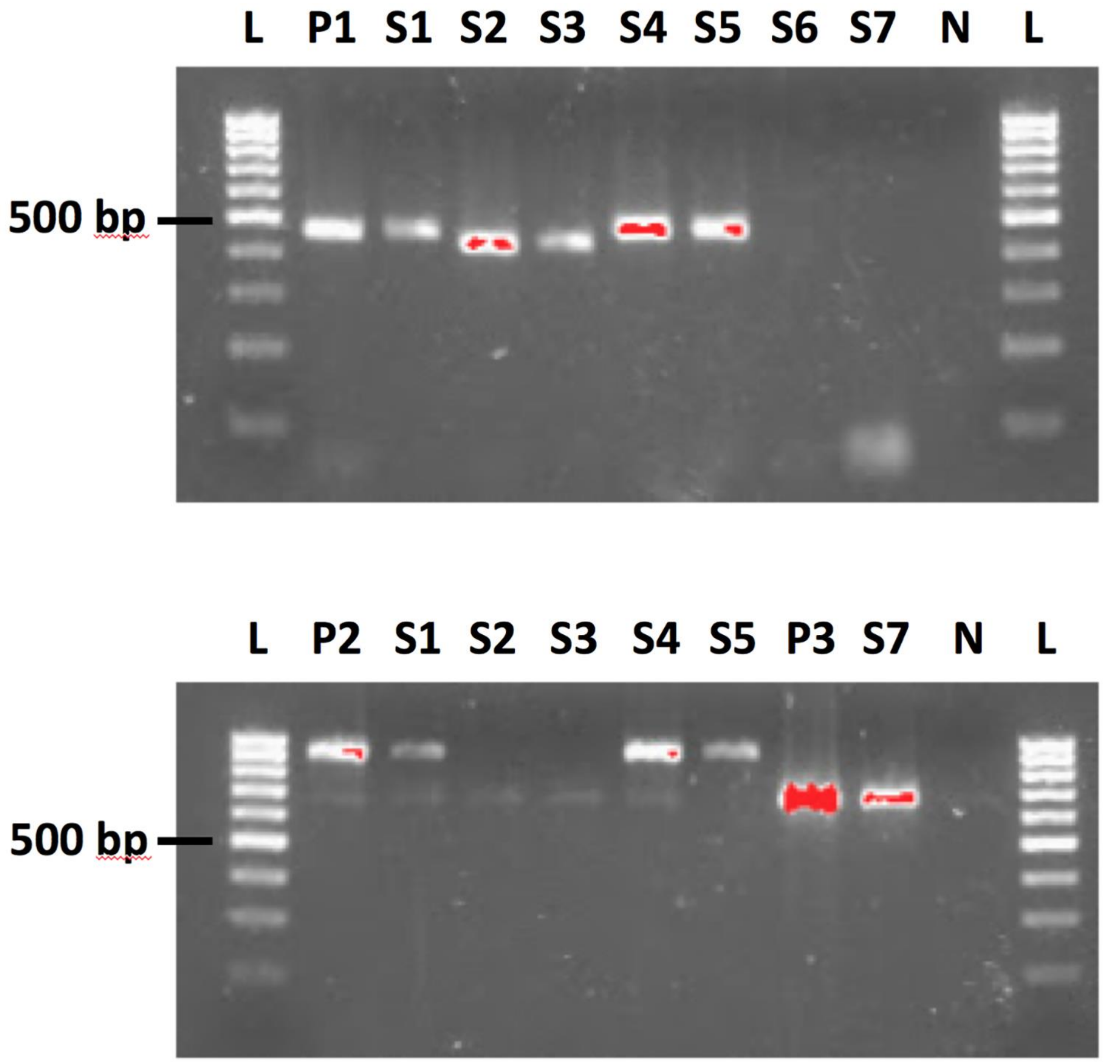

Technical Appendix Figure 3. Representative gel after PCR was performed on amebae DNA extracted from soil isolates. The species of amebae isolated from the soil were used in subsequent laboratory experiments to test how environmental amebae and *Yersinia pestis* interact. Basepair ladder (L), *Acanthamoeba spp.* positive control (P1, 423–551bp) (*3*); *Dictyostelium discoideum*–positive control (P2, 900bp) (*4*); and *Vermamoeba vermiformis*–positive control (P3, 700bp) (5); soil samples (S1–S5).

References

1. Naylor J. Prairie dog towns 2015: Pawnee National Grassland, West Side [map]. 1:100,000. United States Department of Agriculture.: Forest Service. 2016.

2. Lagkouvardos I, Shen J, Horn M. Improved axenization method reveals complexity of symbiotic associations between bacteria and acanthamoebae. Environ Microbiol Rep. 2014;6:383–8. PubMed http://dx.doi.org/10.1111/1758-2229.12162

3. Schroeder JM, Booton GC, Hay J, Niszl IA, Seal DV, Markus MB, et al. Use of subgenic 18S ribosomal DNA PCR and sequencing for genus and genotype identification of acanthamoebae from humans with keratitis and from sewage sludge. J Clin Microbiol. 2001;39:1903–11. PubMed http://dx.doi.org/10.1128/JCM.39.5.1903-1911.2001

4. Charette SJ, Cosson P. Preparation of genomic DNA from *Dictyostelium discoideum* for PCR analysis. Biotechniques. 2004;36:574–5. PubMed

5. Lasjerdi Z, Niyyati M, Haghighi A, Shahabi S, Biderouni FT, Taghipour N, et al. Potentially pathogenic free-living amoebae isolated from hospital wards with immunodeficient patients in Tehran, Iran. Parasitol Res. 2011;109:575–80. PubMed http://dx.doi.org/10.1007/s00436-011-2288-5
